# Occurrence and Molecular Characterization of Abundant *tet*(X) Variants Among Diverse Bacterial Species of Chicken Origin in Jiangsu, China

**DOI:** 10.3389/fmicb.2021.751006

**Published:** 2021-12-20

**Authors:** Yingshan Li, Kai Peng, Yi Yin, Xinran Sun, Wenhui Zhang, Ruichao Li, Zhiqiang Wang

**Affiliations:** ^1^College of Veterinary Medicine, Yangzhou University, Yangzhou, China; ^2^Jiangsu Co-innovation Center for Prevention and Control of Important Animal Infectious Diseases and Zoonoses, Yangzhou, China; ^3^Institute of Comparative Medicine, Yangzhou University, Yangzhou, China

**Keywords:** tigecycline resistance, *tet*(X), plasmids, chickens, whole-genome sequencing

## Abstract

Many novel tigecycline-inactivating enzymes encoded by *tet*(X) variants from different bacteria were discovered since the plasmid-mediated *tet*(X3) and *tet*(X4) genes conferring high-level resistance to tigecycline in Enterobacterales and *Acinetobacter* were reported. However, there have been no comprehensive studies of the prevalence of different *tet*(X) variants in poultry farms. In this study, we collected 45 chicken fecal samples, isolated *tet*(X)-positive strains, and performed antimicrobial susceptibility testing, conjugation assay, whole-genome sequencing, and bioinformatics analysis. A total of 15 *tet*(X)-bearing strains were isolated from 13 samples. Species identification and *tet*(X) subtyping analysis found that the 15 strains belonged to eight different species and harbored four different *tet*(X) variants. Genomic investigation showed that transmission of *tet*(X) variants was associated with various mobile genetic elements, and *tet*(X4) was the most prevalent variant transferred by conjugative plasmids. Meanwhile, we characterized a plasmid co-harboring *tet*(X6) and *bla*_OXA–58_ in *Acinetobacter baumannii*. In summary, we demonstrated that different *tet*(X) variants were widely disseminated in the chicken farming environment and dominated by *tet*(X4). This finding expands the understanding of the prevalence of *tet*(X) among different animal sources, and it was advocated to reduce the usage of antibiotics to limit the emergence and transmission of novel *tet*(X) variants in the poultry industry.

## Introduction

Tigecycline is a broad-spectrum antibiotic of glycylcyclines and is one of the last-resort antibiotics to treat serious infections caused by multidrug-resistant (MDR) Gram-negative bacteria (Sun et al., 2013). The mechanisms of tigecycline resistance were mainly the overexpression of non-specific active efflux pumps or mutations within the drug-binding sites in the ribosome, which were limited by less capability of horizontal transfer among bacteria (Pournaras et al., 2016). However, the emergence and dissemination of plasmid-mediated high-level tigecycline resistance genes *tet*(X3) and *tet*(X4) are bringing formidable threats to public health (He et al., 2019; Sun J. et al., 2019). A variety of *tet*(X) variants containing *tet*(X3.2) (Li et al., 2019), *tet*(X5) (Wang et al., 2019), *tet*(X6) (He et al., 2020; Liu et al., 2020; Peng et al., 2020), and *tet*(X14) (Cheng et al., 2020) have been detected in *Empedobacter*, Enterobacterales, and *Acinetobacter* so far. These widespread *tet*(X) variants will limit treatment options for MDR bacteria infections.

The livestock industry has been a critical reservoir of resistance genes due to the abuse and misuse of antibiotics in animal agriculture. Many clinically significant resistance genes, such as *mcr-1* (Liu et al., 2016), *tet*(X3), and *tet*(X4) (He et al., 2019), were first detected in bacteria of animal origin. According to a retrospective screening project, the prevalence of *tet*(X)-positive isolates of animal source (6.9%) was much higher than that of human source (0.07%) (He et al., 2019). Recent studies also showed that the detection rate of *tet*(X) genes in isolates from animals (Cui et al., 2020; Li et al., 2020b,c) was higher than that from humans (Wang et al., 2019). Hence, it is critical to enrich more information about the animal source associated with *tet*(X)-bearing pathogens. The prevalence of *tet*(X) genes in swine farms and slaughterhouses has been systematically investigated (Li et al., 2020b,c), but the comprehensive molecular characterization of *tet*(X)-bearing bacteria of chicken was unexplored. In this study, we focused on the prevalence of *tet*(X) variants in cultivable bacteria among chicken fecal microbiota and demonstrated that *tet*(X) genes in diverse bacteria are worthy of continuous surveillance among different sources.

## Materials and Methods

### Sample Collection and Bacterial Isolates

A total of 45 chicken fecal samples were collected from a chicken farm in Jiangsu Province, China, in May 2020. We incubated 0.5 g feces in 5 ml of Tryptic Soy Broth (TSB) for 6 h to perform bacteria enrichment. The *tet*(X)-positive isolates were screened by Tryptic Soy Agar (TSA) plates supplemented with tigecycline (4 mg/L) and further identified by PCR using primers previously described (He et al., 2019). The species of all *tet*(X)-positive isolates were determined by 16S rRNA gene sequencing.

### Antimicrobial Susceptibility Testing

The minimum inhibitory concentrations (MICs) of all *tet*(X)-positive isolates were tested by broth microdilution according to Clinical and Laboratory Standards Institute (CLSI) guidelines (CLSI, 2018). *Escherichia coli* ATCC25922 was used for quality control. The resistance breakpoint for tigecycline was interpreted as >0.5 mg/L according to European Committee on Antimicrobial Susceptibility Testing (EUCAST)^1^.

### Conjugation Experiments

In order to verify the transferability of *tet*(X) genes, we conducted conjugation experiments using *E. coli* C600 and a clinical carbapenem-resistant *Acinetobacter baumannii* 5AB as recipients. Briefly, the donor and recipient strains were cultured to the logarithmic growth phase with an optical density at 600 nm (OD_600_) of 0.4 in LB broth, mixed at a ratio of 1:1, and cultured overnight on TSB agar plates at 37°C. For the *tet*(X)-positive *Acinetobacter*, we also conducted the conjugation assay at 30°C. Then, the transconjugants were selected using TSA plates containing tigecycline (2 mg/L) and rifampin (300 mg/L) or meropenem (2 mg/L). And we further confirmed the recovered transconjugants by PCR for *tet*(X) and 16S rRNA genes. The frequencies of conjugation transfer were calculated by the number of transconjugants per recipient.

### Genomic DNA Extraction and Whole-Genome Sequencing

Genomic DNA of *tet*(X)-positive isolates were extracted using FastPure Bacteria DNA Isolation Mini Kit (Vazyme™, Nanjing, China) following the manufacturer’s instruction. The quality and purity of genomic DNA were evaluated by Qubit 4 Fluorometer (Thermo Fisher Scientific™, Hennigsdorf, Germany) and Titertek-Berthold Colibri (Berthold™, Bad Wildbad, Germany). The genomic DNA of all *tet*(X)-positive isolates was subjected to the short-read sequencing (2 × 150 bp) by Illumina Hiseq 2500 platform. According to the assembly result of short-read sequencing, the genomic DNA of isolates with different *tet*(X) genetic contexts was further sequenced by long-read sequencing platform Oxford Nanopore Technologies MinION with a rapid barcoding library preparation strategy.

### Bioinformatics Analysis

*De novo* short-read assembly was performed using SPAdes (Bankevich et al., 2012). The complete bacterial genomes were obtained using a hybrid assembly strategy combining long-read Nanopore and short-read Illumina sequencing data (Wick et al., 2017; Li R. et al., 2018). Antibiotic resistance genes, insertion sequence (IS) elements, and plasmid replicon types were identified by CGE services.^2^ The draft genomes were annotated by Prokka (Seemann, 2014). Functional annotation of the complete genome sequences was annotated automatically using the RAST^3^ and modified manually. Multilocus sequence typing (MLST) of assembled bacterial genomes was performed using the mlst tool^4^ and Pubmlst.^5^ The complete genomes of *tet*(X4)-bearing *E. coli* were downloaded from nr database in the National Center for Biotechnology Information (NCBI). The phylogenetic tree of strains was constructed using Roary and FastTree based on single-nucleotide polymorphisms (SNPs) of core genomes with default parameters (Price et al., 2009; Page et al., 2015). BRIG and Easyfig tools were used to visualize plasmid comparisons (Alikhan et al., 2011) and genetic context comparisons (Sullivan et al., 2011).

## Results

### Characterization of Tigecycline-Resistant Strains

**FIGURE 1:**
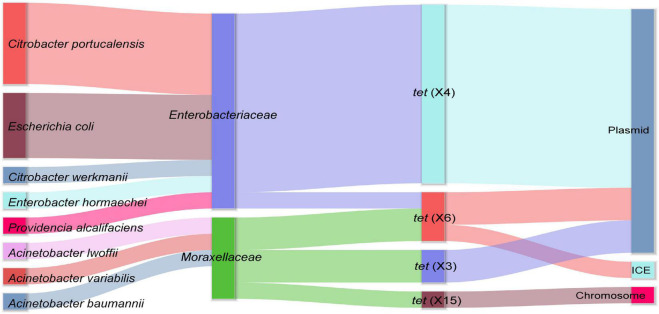
The distribution of *tet*(X)-positive strains and the location of different *tet*(X) variants. The sankey diagram shows the host range diversity and genetic structure features of *tet*(X) variants investigated in this study.

Out of 45 chicken fecal samples, a total of 15 *tet*(X)-positive strains were isolated from 13 samples (13/45, 28.89%). These *tet*(X)-positive strains consisted of eight different species including five *Citrobacter portucalensis*, four *E. coli*, one *Enterobacter hormaechei*, one *Citrobacter werkmanii*, one *Acinetobacter variabilis*, one *Acinetobacter lwoffii*, one *A. baumannii*, and one *Providencia alcalifaciens*. Meanwhile, four different *tet*(X) variants were detected in these strains, containing *tet*(X3), *tet*(X4), and *tet*(X6) reported previously and a novel *tet*(X) variant, designated as *tet*(X15) in another study (Li et al., 2021; Figure 1). Among them, *tet*(X4) carried by Enterobacteriaceae was the most pervasive. Although three different *tet*(X) variants, *tet*(X3), *tet*(X6), and the novel *tet*(X15), were found in *Acinetobacter*, all of them showed low prevalence. Notably, the phenomenon that such a number of *tet*(X) variants were distributed in bacteria with different species within a farm was not observed in other studies. The extensive prevalence of *tet*(X) genes in this chicken farm suggested that poultry may be an important reservoir of *tet*(X), and the *tet*(X) genes are likely to be transmitted to humans through environmental interactions and chicken consumption (Sun et al., 2020).

Although *tet*(X4)-harboring *E. coli* was the most dominant in other research (He et al., 2019; Sun C. et al., 2019; Li et al., 2020a,b; Mohsin et al., 2021), *tet*(X4)-harboring *E. coli* of chicken source was rarely reported previously (Mohsin et al., 2021). To investigate the clonal relationship of *tet*(X4)-harboring *E. coli* between chicken and other sources, the genomes of 26 *tet*(X4)-positive *E. coli* with different hosts including pig, dog, chicken, cow, and human were downloaded from NCBI database and analyzed. Phylogenetic analysis based on the core genome indicated that the prevalence feature of *tet*(X4)-harboring *E. coli* has no clear clonal relationship with their sources (Supplementary Figure 1). It is worth noting that a *tet*(X4)-harboring *E. coli* we detected showed a close relationship with a *tet*(X4)-positive *E. coli* detected in human gut microbiota (Ding et al., 2020; Supplementary Figure 1). Hence, the serious prevalence of *tet*(X) of animal source has a potential threat to human health.

### Antimicrobial Susceptibility Testing and Transfer of Different *tet*(X) Variants

Resistance phenotype analysis found that 15 *tet*(X)-positive strains showed resistance to multiple antibiotics and were all resistant to tigecycline and florfenicol (Table 1). In addition, most of them also conferred resistance to amoxicillin and enrofloxacin, but all strains were still susceptible to meropenem. Subsequently, we analyzed the distribution of resistance genes in *tet*(X)-positive strains according to draft genome sequences constructed by Illumina sequencing data. These strains contained multiple antibiotic resistance genes ranging from 8 to 19 (Supplementary Table 1). Besides, an extended-spectrum beta-lactamase (ESBL) gene *bla*_CTX–M–55_ and a carbapenemase gene *bla*_OXA–58_ were detected in some *tet*(X)-positive strains.

**TABLE 1:** Antimicrobial susceptibility testing (MICs, mg/L) of 15 *tet*(X)-positive strains and their transconjugants.

Strain	Source	Conjugation frequency to C600	ST type	Species	Antimicrobials							
					AMX	ENR	CFF	MEM	CL	KAN	TIG	FFC
SC2-6	Feces	/	284	*Citrobacter portucalensis*	>256	16	1	≤0.25	≤0.25	>128	32	>128
cSC2-6	Transconjugant	2.8 × 10^–9^	/	*Escherichia coli*	>256	16	1	≤0.25	≤0.25	4	16	>128
LHC3	Feces	/	284	*C. portucalensis*	>256	16	1	≤0.25	≤0.25	>128	64	>128
cLHC3	Transconjugant	1.9 × 10^–9^	/	*E. coli*	>256	16	1	≤0.25	≤0.25	8	32	>128
LHC31-1	Feces	/	284	*C. portucalensis*	>256	32	1	≤0.25	≤0.25	>128	32	>128
cLHC31-1	Transconjugant	3.9 × 10^–11^	/	*E. coli*	>256	4	1	≤0.25	≤0.25	4	8	>128
XMY1F802-7	Feces	/	284	*C. portucalensis*	>256	16	0.5	≤0.25	≤0.25	4	64	128
cXMY1F802-7	Transconjugant	3.9 × 10^–11^	/	*E. coli*	>256	4	0.5	≤0.25	≤0.25	4	64	>128
XM10F302-7	Feces	/	284	*C. portucalensis*	>256	32	1	≤0.25	≤0.25	>128	128	>128
cXM10F302-7	Transconjugant	1.4 × 10^–10^	/	*E. coli*	>256	32	1	≤0.25	≤0.25	≤0.25	64	>128
LHC5-1	Feces	/	novel	*Citrobacter werkmanii*	>256	16	16	≤0.25	≤0.25	32	64	>128
cLHC5-1	Transconjugant	1.3 × 10^–9^	/	*E. coli*	>256	0.5	≤0.25	≤0.25	≤0.25	≤0.25	32	>128
XM3F402-1	Feces	/	93	*E. coli*	>256	4	≤0.25	≤0.25	≤0.25	8	32	>128
cXM3F402-1	Transconjugant	4 × 10^–11^	/	*E. coli*	>256	0.5	≤0.25	≤0.25	≤0.25	4	16	>128
XMC1F102-2	Feces	/	93	*E. coli*	>256	4	≤0.25	≤0.25	≤0.25	4	32	>128
cXMC1F102-2	Transconjugant	NA	/	*E. coli*	>256	4	≤0.25	≤0.25	≤0.25	4	16	>128
XM3F402-7	Feces	/	1,286	*E. coli*	>256	4	≤0.25	≤0.25	≤0.25	>128	64	>128
cXM3F402-7	Transconjugant	4 × 10^–11^	/	*E. coli*	>256	4	≤0.25	≤0.25	≤0.25	≤0.25	16	>128
XM7F102	Feces	/	155	*E. coli*	>256	32	>64	≤0.25	≤0.25	8	32	>128
cXM7F102	Transconjugant	NA	/	*E. coli*	>256	2	≤0.25	≤0.25	≤0.25	4	16	>128
LHC3-2	Feces	/	327	*Enterobacter hormaechei*	>256	4	32	≤0.25	≤0.25	8	32	>128
cLHC3-2	Transconjugant	NA	/	*E. coli*	>256	4	≤0.25	≤0.25	≤0.25	4	16	128
LHC2-1	Feces	/	/	*Providencia alcalifaciens*	>256	8	32	≤0.25	>128	>128	>128	>128
XM9F202-2	Feces	/	/	*Acinetobacter variabilis*	2	4	4	≤0.25	≤0.25	>128	32	>128
XMC5X702	Feces	/	/	*Acinetobacter lwoffii*	128	0.5	1	≤0.25	≤0.25	8	32	>128
LHC22-2	Feces	/	1,459	*Acinetobacter baumannii*	>256	32	32	1	≤0.25	16	64	128

*NA, not available. The transfer frequencies of these samples were too low to be calculated accurately.*

*MICs, minimum inhibitory concentrations; ST, sequence typing; AMX, amoxicillin; ENR, enrofloxacin; CFF, ceftiofur; MEM, meropenem; CL, colistin; KAN, kanamycin; TIG, tigecycline; FFC, florfenicol.*

To investigate the transmissibility of different *tet*(X) variants, all strains were performed by conjugation assay. All *tet*(X4) genes in this study were successfully transferred into the recipient *E. coli* C600 with low frequencies, resulting in resistance to tigecycline in transconjugants. The remaining *tet*(X)-positive strains failed in conjugation assay. The higher horizontal transfer percentage of *tet*(X4) might explain its high prevalence.

### The Genetic Contexts of *tet*(X) Variants

**FIGURE 2:**
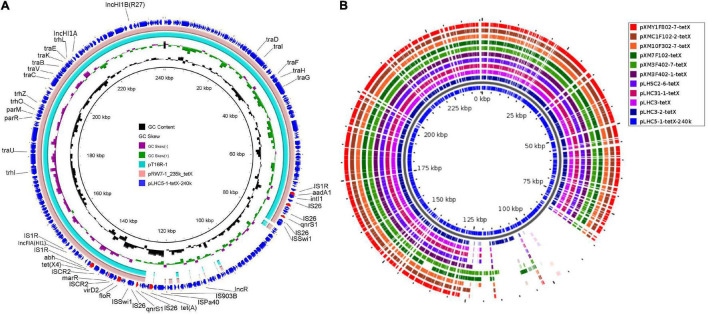
Structure analysis of *tet*(X4)-bearing plasmids. **(A)** Comparison analysis of the plasmid pLHC5-1-tetX-240k with other similar plasmids including pRW7-1_235k_tetX (GenBank accession_number: MT219825) and pT16R-1 (CP046717). **(B)** Structure features of *tet*(X4)-bearing plasmids carried by Enterobacteriaceae in this study. The structural diversity of these plasmids existed within a multidrug-resistant (MDR) region. Resistance genes in plasmid pLHC5-1-tetX-240k were highlighted in red arrows. The reference sequence in panel **(B)** is pLHC5-1-tetX-240k, and colored circles indicate the sequences in draft genomes, which are mapped to the reference sequence.

**FIGURE 3:**
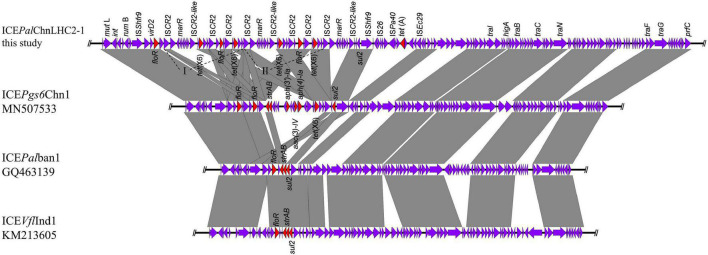
Linear comparison of the *tet*(X6)-bearing integrative and conjugative element (ICE) ICE*Pal*ChnLHC2-1 with other similar ICEs. The multidrug-resistant (MDR) region encoding *tet*(X6) was inserted into variable region III conserved in ICEs.

In order to investigate the genetic contexts of different *tet*(X) variants, five strains (one *C. werkmanii* LHC5-1, one *P. alcalifaciens*, and three *Acinetobacter*) were performed with Nanopore long-read sequencing to obtain complete genomes together with short-read data (Supplementary Table 1). Genetic analysis of strain LHC5-1 found that *tet*(X4) gene was located in a 240-kb IncFIA(HI1)/IncHI1A/IncHI1B(R27)/IncR hybrid plasmid, named pLHC5-1-tetX-240k. Many plasmids with a similar structure to pLHC5-1-tetX-240k were found in the NCBI nr database (Figure 2A), and most of these plasmids were positive for *tet*(X4) and harbored by *E. coli*. The emergence of *tet*(X4)-bearing IncFIA(HI1)/IncHI1A/IncHI1B(R27)/IncR plasmid in *Citrobacter* spp. exacerbated the transmission of *tet*(X4) among different species of bacteria. Comparative analysis of plasmid pLHC5-1-tetX-240k and other similar hybrid plasmids found that a ca. 190-kb backbone region with replicons IncFIA(HI1)/IncHI1A/IncHI1B(R27) in these hybrid plasmids was conserved (Figure 2A). Some small plasmids with replicons IncX1, IncX4, and IncR could integrate into a hybrid plasmid with replicons IncFIA(HI1)/IncHI1A/IncHI1B(R27) and form larger and more complex plasmids, such as pRW7-1_235k_tetX (Li et al., 2020b). Subsequently, we investigated the genetic feature of *tet*(X4) in other strains in this study. The result showed that all *tet*(X4) genes in this study were carried by plasmids with a similar backbone to pLHC5-1-tetX-240k and located in a conserved ca. 190-kb region harboring IncFIA(HI1)/IncHI1A/IncHI1B(R27) plasmid replicons (Figure 2B). In addition, we found that these hybrid plasmids were widely distributed in different species of bacteria. Therefore, the diffusion of *tet*(X4) was strongly associated with the IncFIA(HI1)/IncHI1A/IncHI1B(R27) hybrid plasmids and their evolved complex plasmids. Apart from these *tet*(X4)-bearing plasmids in Enterobacteriaceae, one *tet*(X6) gene was detected in a *P. alcalifaciens* of Enterobacteriaceae for the first time. *tet*(X6) gene was located in variable region III (VRIII) of a chromosomal integrative and conjugative element (ICE), designated as ICE*Pal*ChnLHC2-1. A total of four *tet*(X6) genes were detected in VRIII within two tandem repeat units (Figure 3). Although tandem repeats of different *tet*(X) variants were frequently observed, two *tet*(X6) in one repeat unit have not been reported. The molecular mechanism of *tet*(X) tandem repeat deserved further investigations. Then, we searched for homologous ICEs with ICE*Pal*ChnLHC2-1 in the NCBI database, and three *tet*(X)-negative ICEs from *Proteus genomosp*, *P. alcalifaciens*, and *Vibrio fluvialis* were downloaded and compared. The four ICEs showed high similarity with each other, which implied that they originated from one ancestor and were popular because of horizontal transfer between different bacterial chromosomes. Meanwhile, we observed an evolution of genetic context in VRIII of the four ICEs, which was a manifestation of the adaptation of bacteria to changes in the external environment.

**FIGURE 4:**
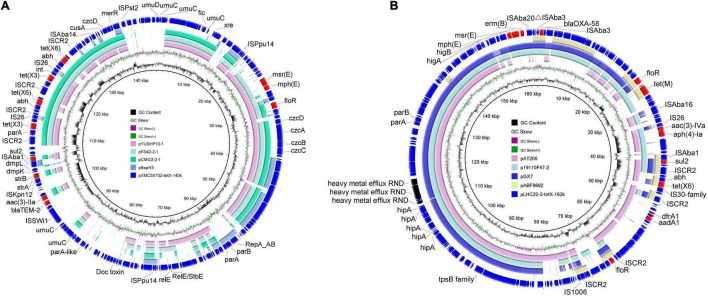
Circular comparisons between *tet*(X)-positive plasmids of *Acinetobacter* origin in this study and similar plasmids in the National Center for Biotechnology Information (NCBI) database. **(A)** Comparative analysis of pXMC5X702-tetX-145k with four closely related plasmids including pBspH3 (GenBank accession_number: CP055285), pCMG3-2-1 (CP044446), pFS42-2-1 (CP046596), and pYUSHP10-1 (MT107270). pXMC5 × 102-tetX-145k was used as the reference plasmid. **(B)** Comparative analysis of pLHC22-2-tetX-162k with four closely related plasmids including pABF9692 (CP048828), pGX7 (CP071772), p19110F47-2 (CP046044), and pAT205 (CP048015). Plasmid pBspH3 in panel **(A)** shared the same replicon gene with plasmid pXMC5X702-tetX-145k. Plasmid pABF9692 co-harbored *tet*(X6) and *bla*_*OXA–*58_ but shared limited homologous regions with plasmid pLHC22-2-tetX-162k.

Although only three *tet*(X)-positive strains belonging to Moraxellaceae were identified, complex genetic contexts of *tet*(X) variants were found in the three strains. Co-occurrence of two different *tet*(X) variants in one strain was detected in strains XM9F202-2 and XMC5X702. A plasmid-mediated *tet*(X3) and a chromosomal novel *tet*(X) variant, designated as *tet*(X15), were found in *A. variabilis* XM9F202-2, which has been investigated in detail in our previous study (Li et al., 2021). In *A. lwoffii* XMC5X702, two different *tet*(X) variants corresponded to *tet*(X3) and *tet*(X6). Genetic analysis found that *tet*(X3) and *tet*(X6) were located on a 145-kb plasmid pXMC5X702-tetX-145k with unknown replicon types. Multiple plasmids co-harboring *tet*(X3) and *tet*(X6) carried by *Acinetobacter* were found in the nr database, and they share more than 50% coverage and more than 95% identify to pXMC5X702-tetX-145k (Figure 4A). However, the replicon gene in pXMC5X702-tetX-145k differed from those plasmids co-harboring *tet*(X3) and *tet*(X6). The plasmids that harbor the same replicon as pXMC5X702-tetX-145k showed low identity to pXMC5X702-tetX-145k. Hence, the structure of pXMC5X702-tetX-145k was novel, and it enriched the profile of *tet*(X)-bearing plasmids in *Acinetobacter*. *tet*(X6) gene in *A. baumannii* LHC22-2 was carried by a 162-kb plasmid pLHC22-2-tetX-162k. What is noteworthy is that a carbapenemase gene *bla*_OXA–58_ was found in the *tet*(X6)-bearing plasmid. Although many plasmids co-harboring *tet*(X3) and *bla*_OXA–58_ have been reported in other species of *Acinetobacter* (Cui et al., 2020; Ma et al., 2020), *tet*(X6)- and *bla*_OXA–58_-bearing plasmid was rarely reported (Zheng et al., 2020). As a clinically critical opportunistic pathogen, the emergence of carbapenem- and tigecycline-resistant *A. baumannii* poses a great threat to public health. Phenotype analysis of antimicrobial resistance showed that LHC22-2 was resistant to imipenem but susceptible to meropenem. The expression of *bla*_OXA–58_ could be enhanced by an intact upstream IS*Aba3* and result in resistance to meropenem (Hamidian and Nigro, 2019), but IS*Aba3* in plasmid pLHC22-2-tetX-162k was truncated. Subsequently, one plasmid pABF9692 co-harboring *tet*(X6)- and *bla*_OXA–58_ from *A. baumannii* and three plasmids with different sizes from *Acinetobacter towneri* showing similar backbone with pLHC22-2-tetX-162k were retrieved from the nr database and analyzed (Figure 4B). Notably, the backbone of pABF9692 was different with pLHC22-2-tetX-162k. In contrast, the three plasmids co-harboring *tet*(X3) and *bla*_OXA–58_ showed similar backbone with pLHC22-2-tetX-162k (Figure 4B). Two different *tet*(X) variants, *tet*(X3) and *tet*(X6), were detected in these plasmids, which indicated that such plasmids played a vital role in capturing and propagating the *tet*(X) genes.

### The Core Genetic Structures of *tet*(X) Variants in This Study

**FIGURE 5:**
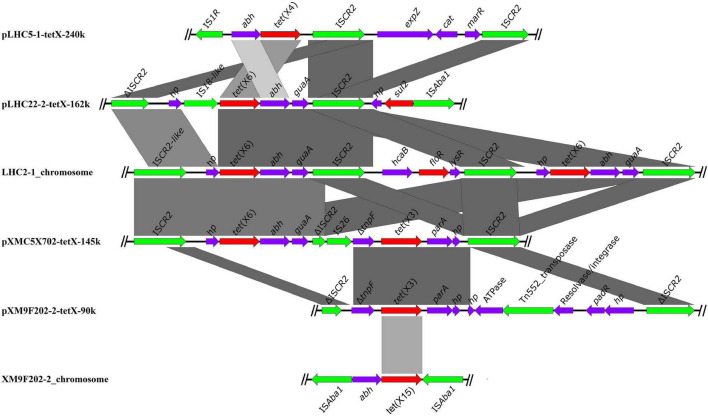
The core genetic structures of *tet*(X) investigated in this study. The resistance genes are shown as red arrows, and the mobile elements are shown as green arrows.

The different *tet*(X) variants were harbored by various genetic structures and distributed in different bacteria in the chicken farm. However, IS*CR2* was always associated with different *tet*(X) variants except for the novel *tet*(X15) (Figure 5). This phenomenon was consistent with previous studies (He et al., 2019; Li et al., 2020b,c), implying that IS*CR2* was a major driving factor for the dissemination of *tet*(X) variants. We also found many other IS elements in the surroundings of different *tet*(X) variants, such as IS*Aba1* in the downstream of *tet*(X6) in plasmid pLHC22-2-tetX-162k and IS*26* in the upstream of *tet*(X3) in plasmid pXMC5X702-tetX-145k. These IS elements will probably be involved in the transfer of *tet*(X) variants and hereby have evolved novel genetic context of *tet*(X) variants. Apart from IS*CR2*-associated *tet*(X)-bearing genetic contexts, we identified a novel *tet*(X15) located in an IS*Aba1*-bound composite transposon Tn*6866* (Li et al., 2021). The IS*Aba1* in the composite transposon Tn*6866* is directly responsible for the movement of *tet*(X15), which differs from that of *tet*(X6). Therefore, monitoring the genetic context of *tet*(X) variants is important for understanding their transmission and evolution destiny.

## Discussion

The emergence of high-level tigecycline resistance genes *tet*(X3) and *tet*(X4) has caused great concern throughout the world. A large number of *tet*(X) variants, from *tet*(X3) to *tet*(X44), were identified from different bacteria in humans and animals within 2 years (Wang et al., 2019; Cheng et al., 2020; Gasparrini et al., 2020; Peng et al., 2020; Zheng et al., 2020; Umar et al., 2021). The current outbreak and widespread situation of *tet*(X) is rapidly diminishing the effectiveness of tetracycline antibiotics, including tigecycline and the US Food and Drug Administration (FDA) newly approved eravacycline and omadacycline. Tetracyclines have been used in livestock farms for many years in China. However, few studies investigated the epidemiological and genetic features of *tet*(X) in livestock farms, with limited research focusing on the *tet*(X)-bearing Enterobacterales or *Acinetobacter* (Cui et al., 2020; Li et al., 2020c). Meanwhile, the prevalence of *tet*(X) in bacteria of chicken origin has not been investigated fully to date. In this work, we systematically explored the distribution and genetic characteristics of different *tet*(X) variants and their host bacteria in a chicken farm. We found that the prevalence of mobilizable *tet*(X4) was the highest and more worrisome than that of other variants. Apart from *E. coli*, *Citrobacter* spp. was also an emerging host for *tet*(X4). The high prevalence of *tet*(X4) might be associated with their host plasmids. Although only three *tet*(X)-positive strains belonging to *Acinetobacter* spp. were identified, three different *tet*(X) variants were identified in them. The epidemic pattern of *tet*(X) in *Acinetobacter* differed from that in Enterobacterales, and the relationship between them warrants further investigations.

Genetic analysis found that plasmids are an important vector for the dissemination of *tet*(X). However, some chromosomal mobile elements, such as ICEs and transposons, also contribute to the transfer of *tet*(X). According to transfer experiments, all *tet*(X4)-positive plasmids in Enterobacterales could transfer to *E. coli* C600, and the other *tet*(X)-bearing genetic structures in Enterobacterales and *Acinetobacter* failed to transfer in conjugation assay. The phenomenon explained the high prevalence of *tet*(X4) in the chicken farm and demonstrated that the prevalence of *tet*(X) genes was positively related to the horizontal transferability of their vectors within specific bacterial hosts. Notably, the transmission of *tet*(X4) was associated with various plasmids reported in our previous study (Li et al., 2020b). In this study, we first noticed that serious prevalence of *tet*(X4) in different bacteria mediated by IncFIA(HI1)/IncHI1A/IncHI1B(R27) plasmids occurred in the chicken farm. Currently, the worldwide dissemination of critical resistance genes was possible with the help of some common types of plasmids, such as *bla*_NDM–5_-positive IncX3 plasmid (Li X. et al., 2018; Liu et al., 2019; Zhao et al., 2021) and *mcr*-1-positive IncI2 plasmid (Elbediwi et al., 2019; Gelbicova et al., 2019; Lu et al., 2020). Hence, the emergence of *tet*(X4)-positive common plasmids with high mobility might cause an increasing prevalence of *tet*(X4). In addition, we found that all *tet*(X)-positive plasmids in *Acinetobacter* in the chicken farm had no ability of horizontal transfer, which is consistent with the previous reports (Cui et al., 2020; Ma et al., 2020; Wang et al., 2020). Genetic structure analysis found that those *tet*(X) genes in plasmids harbored by *Acinetobacter* were adjacent to IS*CR2*, indicating that IS*CR2-*mediated mobilization of *tet*(X) also deserved concerns among bacteria of different genus.

In conclusion, we comprehensively investigated the prevalence of *tet*(X) in a chicken farm first and identified multiple *tet*(X) variants from diversified bacteria. The prevalence of *tet*(X4) in the chicken farm was mainly determined by their host plasmids. The *Acinetobacter* spp. is an important reservoir for other *tet*(X) variants. Apart from IS*CR2*, IS*Aba1* might also be an important element for the mobilization of *tet*(X). Therefore, we propose that effective measures should be formulated to decelerate the dissemination of *tet*(X) in animal- and human-associated environments.

## Data Availability Statement

The sequences obtained in this article have been deposited in the GenBank database under BioProject number: PRJNA750704.

## Author Contributions

RL and ZW conceived and designed the experiments, and manuscript reviewing and editing. YL and KP conducted the experiments, analyze the data, and wrote the draft. YY, XS, and WZ conducted long-read sequencing and bioinformatics analysis. All authors read and approved the final manuscript.

## Conflict of Interest

The authors declare that the research was conducted in the absence of any commercial or financial relationships that could be construed as a potential conflict of interest.

## Publisher’s Note

All claims expressed in this article are solely those of the authors and do not necessarily represent those of their affiliated organizations, or those of the publisher, the editors and the reviewers. Any product that may be evaluated in this article, or claim that may be made by its manufacturer, is not guaranteed or endorsed by the publisher.

## Footnotes

1
http://www.eucast.org/clinical_breakpoints/

2
https://cge.cbs.dtu.dk/services/

3
http://rast.nmpdr.org/

4
https://github.com/tseemann/mlst

5
https://pubmlst.org/
