# Supplementary material for: Occurrence and Molecular Characterization of Abundant tet(X) Variants Among Diverse Bacterial Species of Chicken Origin in Jiangsu, China
Source: Front Microbiol. 2021 Dec 20;12:751006. doi: 10.3389/fmicb.2021.751006 (PMC8723793; doi:10.3389/fmicb.2021.751006)
Supplement: Supplementary file 1 [file Data_Sheet_1.docx]

**Supplementary Data**


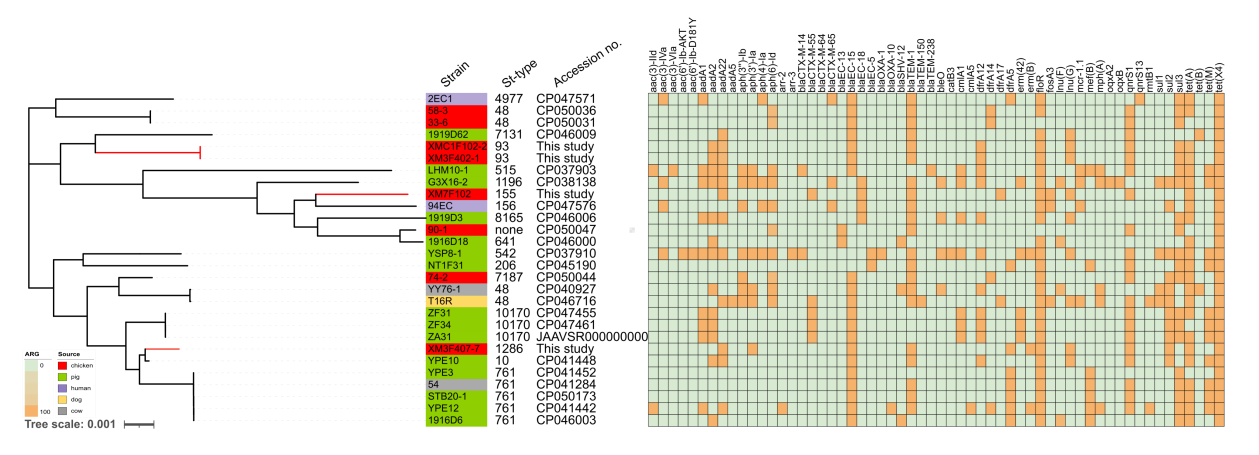
**Figure S1. Phylogentic tree of *tet*(X4) positive *E. coli* strains ofdifferent sources based on SNPs of core genome.**Closed related strains were clustered into one clonal group.ARG indicated antibiotic resistance gene. Apart from *tet*(X4), most of these strains also harbored *bla*_TEM-1_, *floR*, *qnrS1*, *sul3* and *tet*(A).

**Supplementary Table 1. Basic information of the 15*tet*(X) positive strains in this study.**

| Sample | Strain | Species | Assembly Method | Sequencing Technology | Resistance genes | Locations of *tet*(X) |
| --- | --- | --- | --- | --- | --- | --- |
| SC2 | SC2-6 | *Citrobacter portucalensis* | SPAdes | Illumina | *aac(3)*-*IVa*, *aadA2*, *aadA22*, *aph(3')-Ia*, *aph(4)-Ia*, *bla*_CMY-63_, *bla*_TEM-1_, *dfrA12*, *floR*, *lnu(G)*, *qnrB54*, *qnrS1*, *sul2*, *tet*(D), *tet*(X4) | Plasmid |
| LHC3 | LHC3 | *Citrobacter portucalensis* | SPAdes | Illumina | *aac(3)-IVa*, *aadA2*, *aadA22*, *aph(3')-Ia*, *aph(4)-Ia*, *bla*_CMY-63_, *bla*_TEM-1_, *dfrA12*, *floR*, *lnu(G)*, *qnrB54*, *qnrS1*, *sul2*, *tet*(D), *tet*(X4) | Plasmid |
| LHC31 | LHC31-1 | *Citrobacter portucalensis* | SPAdes | Illumina | *aac(3)*-*IVa*, *aadA2*, *aadA22*, *aph(3')-Ia*, *aph(4)-Ia*, *bla*_CMY-63_, *bla*_TEM-1_, *dfrA12*, *floR*, *lnu(G)*, *qnrB54*, *qnrS1*, *sul2*, *tet*(D), *tet*(X4) | Plasmid |
| XMY1F802 | XMY1F802-7 | *Citrobacter portucalensis* | SPAdes | Illumina | *aadA22*, *bla*_CMY-63_, *bla*_TEM-1_, *floR*, *lnu(G)*, *qnrB54*, *qnrS1*,*tet*(X4) | Plasmid |
| XM10F302 | XM10F302-7 | *Citrobacter portucalensis* | SPAdes | Illumina | *aac(3)*-*IVa*, *aadA2*, *aadA22*, *aph(3')-Ia*, *aph(4)-Ia*, *bla*_CMY-63_, *bla*_TEM-1_, *dfrA12*, *floR*, *lnu(G)*, *qnrB54*, *qnrS1*, *sul2*, *tet*(D), *tet*(X4) | Plasmid |
| LHC5 | LHC5-1 | *Citrobacter werkmanii* | Unicycler | Illumina, Oxford Nanopore MinION | *aac(6')-Ib*, *aadA16*, *aadA22*, *aph(3'')-Ib*, *aph(6)-Id*, *arr-3*, *bla*_CMY-159_, *bla*_TEM-1_, *dfrA27*, *floR*, *lnu(G)*, *qnrB12*, *qnrS1*, *sul1*, *sul2*, *tet*(A), *tet*(X4) | Plasmid |
| XM3F402 | XM3F402-1 | *Escherichia coli* | SPAdes | Illumina | *aadA2*, *aadA22*, *bla*_TEM-1_, *bla*_EC-15_, *dfrA12*, *floR*, *lnu(G)*, *qnrB54*, *qnrS1*, *tet*(D), *tet*(X4) | Plasmid |
| XMC1F102 | XMC1F102-2 | *Escherichia coli* | SPAdes | Illumina | *aadA2*, *aadA22*, *bla*_TEM-1_, *bla*_EC-15_, *dfrA12*, *floR*, *lnu(G)*, *qnrB54*, *qnrS1*, *tet*(D), *tet*(X4) | Plasmid |
| XM3F402 | XM3F402-7 | *Escherichia coli* | SPAdes | Illumina | *aadA22*, *aph(3')-Ia*, *bla*_EC-15_, *bla*_TEM-1_, *dfrA15*, *erm(B)*, *floR*, *lnu(G)*, *qnrS1*, *sul2*, *tet*(A), *tet*(X4) | Plasmid |
| XM7F102 | XM7F102 | *Escherichia coli* | SPAdes | Illumina | *aadA22*, *bla*_CTX-M-55_, *bla*_EC-18_, *bla*_TEM-1_, *dfrA17*, *floR*, *fosA3*, *lnu(G)*, *qnrS1*, *tet*(A), *tet*(X4) | Plasmid |
| LHC3 | LHC3-2 | *Enterobacter hormaechei* | SPAdes | Illumina | *aac(3)*-*IVa*, *aadA2*, *aadA22*, *aph(3'')-Ib*, *aph(4)-Ia*, *aph(6)-Id*, *bla*_ATC-23_, *bla*_TEM-1_, *dfrA12*, *floR*, *fosA*, *lnu(F)*, *lnu(G),oqxA10*, *oqxB5*, *qnrS1*, *sul2*, *tet*(B), *tet*(X4) | Plasmid |
| LHC2 | LHC2-1 | *Providencia alcalifaciens* | Unicycler | Illumina, Oxford Nanopore MinION | *aac(3)-IVa*, *aac(6')-Ib*, *aadA2*, *aadA5*, *aph(3')-Ia*, *aph(4)-Ia*, *arr-3*, *bla*_OXA-1_, *catB3*, *dfrA1*, *dfrA17*, *qnrD1*, *sul1*, *sul2*, *tet*(A), *tet*(B), *tet*(X6) | Chromosome |
| XM9F202 | XM9F202-2 | *Acinetobacter variabilis* | Unicycler | Illumina, Oxford Nanopore MinION | *aac(3)-IId*, *aadA1*, *aph(3'')-Ib*, *aph(3')-Ia*, *aph(3')-VI*, *aph(6)-Id*, *dfrA1*, *floR*, *lnu(G)*, *mph(E)*, *msr(E)*, *sat2*, *sul2*, *tet*(M), *tet*(X3), *tet*(X15) | Plasmid and Chromosome |
| XMC5X702 | XMC5X702 | *Acinetobacter lwoffii* | Unicycler | Illumina, Oxford Nanopore MinION | *aac(3)-IIe*, *ant(2'')-Ia*, *aph(6)-Id*, *bla*_TEM-2_, *dfrA20*, *dfrA36*, *floR*, *lnu(G)*, *mph(E)*, *msr(E)*, *sul2*, *tet*(X3), *tet*(X6) | Plasmid |
| LHC22 | LHC22-2 | *Acinetobacter baumannii* | Unicycler | Illumina, Oxford Nanopore MinION | *aac(3)-IVa*, *aadA1*, *aph(3'')-Ib*, *aph(4)-Ia*, *aph(6)-Id*, *bla*_ADC-166_, *bla*_OXA-554_, *bla*_OXA-58_, *dfrA1*, *erm(B)*, *mph(E)*, *msr(E)*, *sat2*, *sul2*, *tet*(39), *tet*(M), *tet*(X6) | Plasmid |
